# Role of Propriospinal Neurons in Control of Respiratory Muscles and Recovery of Breathing Following Injury

**DOI:** 10.3389/fnsys.2019.00084

**Published:** 2020-01-17

**Authors:** Victoria N. Jensen, Warren J. Alilain, Steven A. Crone

**Affiliations:** ^1^Neuroscience Graduate Program, University of Cincinnati College of Medicine, Cincinnati, OH, United States; ^2^Spinal Cord and Brain Injury Research Center, University of Kentucky College of Medicine, Lexington, KY, United States; ^3^Department of Neuroscience, University of Kentucky College of Medicine, Lexington, KY, United States; ^4^Division of Neurosurgery, Cincinnati Children’s Hospital Medical Center, Cincinnati, OH, United States; ^5^Division of Developmental Biology, Cincinnati Children’s Hospital Medical Center, Cincinnati, OH, United States; ^6^Department of Neurosurgery, University of Cincinnati College of Medicine, Cincinnati, OH, United States

**Keywords:** neuroplasticity, spinal cord injury (SCI), interneurons, breathing, central pattern generation (CPG)

## Abstract

Respiratory motor failure is the leading cause of death in spinal cord injury (SCI). Cervical injuries disrupt connections between brainstem neurons that are the primary source of excitatory drive to respiratory motor neurons in the spinal cord and their targets. In addition to direct connections from bulbospinal neurons, respiratory motor neurons also receive excitatory and inhibitory inputs from propriospinal neurons, yet their role in the control of breathing is often overlooked. In this review, we will present evidence that propriospinal neurons play important roles in patterning muscle activity for breathing. These roles likely include shaping the pattern of respiratory motor output, processing and transmitting sensory afferent information, coordinating ventilation with motor activity, and regulating accessory and respiratory muscle activity. In addition, we discuss recent studies that have highlighted the importance of propriospinal neurons for recovery of respiratory muscle function following SCI. We propose that molecular genetic approaches to target specific developmental neuron classes in the spinal cord would help investigators resolve the many roles of propriospinal neurons in the control of breathing. A better understanding of how spinal circuits pattern breathing could lead to new treatments to improve breathing following injury or disease.

## Introduction

Respiratory failure is the leading cause of death in spinal cord injury (SCI) (Berlly and Shem, 2007; Berlowitz et al., 2016). Connections between brainstem respiratory centers and respiratory motor neurons in the spinal cord are disrupted following injury, leading to loss of respiratory drive. Since respiratory motor neurons also receive excitatory and inhibitory inputs from propriospinal neurons, spinal circuits may serve as substrates to improve breathing following injury (Lane, 2011; Lee and Fuller, 2011; Marchenko et al., 2015; Zholudeva et al., 2018b). For the purposes of this review, we refer to propriospinal neurons as neurons whose cell bodies are located in the spinal cord but do not project outside the central nervous system. They may have short segmental projections, long multi-segmental spinal projections, and/or projections to the brainstem. Propriospinal neurons appear to contribute to patterning respiratory motor output to ensure efficient and appropriate ventilation over a wide range of behaviors and physiological conditions, whereas brainstem neurons are primarily responsible for generating the three phases of respiratory rhythm (inspiration, post-inspiration, and active expiration; Del Negro et al., 2018; Ramirez and Baertsch, 2018). This review will summarize some of the roles of spinal circuits in the control of breathing in healthy animals, as well as their potential roles in adapting to injury to maintain ventilation. We will also discuss how modern tools to label or manipulate specific developmental neuron classes could help investigators further probe the role of propriospinal neurons in the control of breathing.

## Propriospinal Neurons Pattern Respiratory Motor Activity

**Figure 1:**
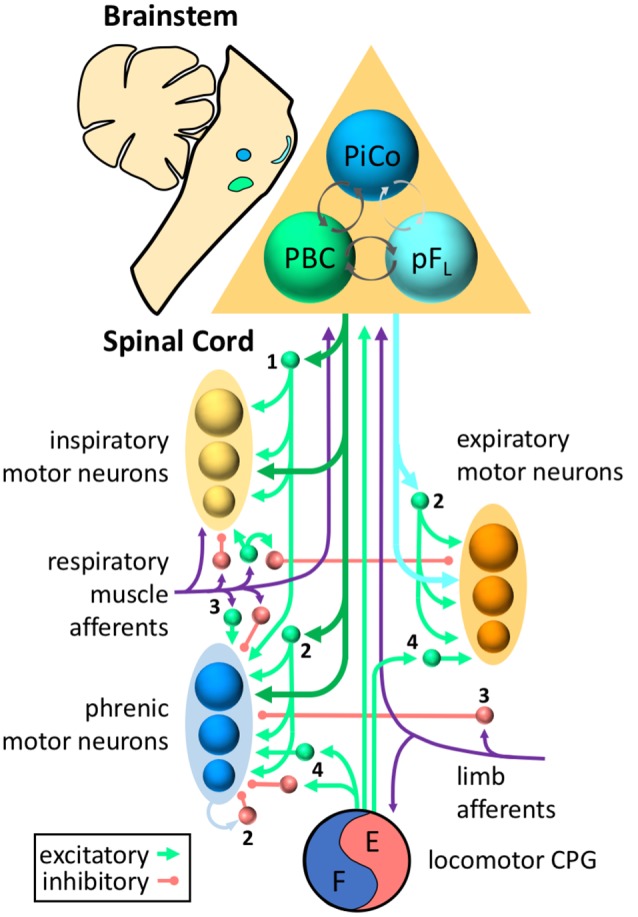
Roles for propriospinal neurons in control of respiratory muscles. Respiratory rhythm is generated by three oscillators located in discrete regions of the brainstem—the pre-Bötzinger complex (PBC, green) for inspiration, the lateral parafacial region (pFL, turquoise) for active expiration, and the post-inspiratory complex (PiCo, blue) for the post-inspiratory phase of breathing. Excitatory and inhibitory interactions (with inhibition typically dominating) between the three oscillators ensure coordination of each phase (dark gray arrows = known connections, light gray arrows = hypothesized connections). The inspiratory drive is transmitted from the brainstem to the spinal cord *via* bulbospinal neurons of the rostral ventral respiratory group (dark green arrows), whereas expiratory drive is transmitted *via* bulbospinal neurons of the caudal ventral respiratory group (turquoise arrows). Bulbospinal pathways can provide drive directly to phrenic motor neurons (blue), other inspiratory motor neurons (yellow, e.g., external intercostal, scalene, etc.), or expiratory motor neurons (orange, e.g., abdominal muscles). In addition, bulbospinal pathways can activate propriospinal neurons (excitatory = green, inhibitory = red) that modulate respiratory motor activity. Sensory afferents (purple) from respiratory muscles or limb muscles can project to propriospinal neurons as well as the brainstem. For clarity, not all neurons/connections are shown. The roles of propriospinal neurons in controlling breathing include: **(1)** coordinating different respiratory motor pools; **(2)** patterning the activity of respiratory motor neurons within the same motor pool; **(3)** processing and transmitting afferent input; and **(4)** coordinating locomotor and respiratory activity.

Breathing requires a complex system of muscles to draw air into the lungs, maintain an open airway, and subsequently expel air out of the lungs. This requires coordination between different inspiratory and expiratory muscles, as well as precise control of the amplitude and timing of respiratory muscle activity. Propriospinal neurons play important roles in patterning this activity. Although more work is needed to better define the functional roles of propriospinal neurons in the control of breathing, there is evidence that these roles include shaping the pattern of respiratory motor output, processing and transmitting sensory afferent information, and coordinating ventilation with motor activity (Figure 1).

### Propriospinal Neurons Shape the Pattern of Respiratory Motor Output

Intraspinal stimulation studies indicate that a complex propriospinal network located throughout the cervical and thoracic cord can control the activity of inspiratory muscles (Sunshine et al., 2018). Respiratory propriospinal neurons have been described in the spinal cord of the cat, dog, rat, mouse, and human. They are located in cervical, thoracic and lumbar spinal segments and are distributed throughout the ventral horn, intermediate laminae, and dorsal horn (for reviews, see Lane, 2011; Ikeda et al., 2017; Zaki Ghali et al., 2019). These neurons include pre-phrenic neurons in C3–6 (near the phrenic nucleus, whose motor neurons innervate the diaphragm) as well as high cervical spinal cord neurons in C1-2 (Oku et al., 2008; Okada et al., 2009; Jones et al., 2012). Inspiratory and expiratory neurons are also found in the thoracic cord and it has been estimated that they outnumber bulbospinal respiratory neurons by 10 to 1 (Kirkwood et al., 1988). Both excitatory and inhibitory respiratory neurons have been described (Saywell et al., 2011; Iizuka et al., 2016, 2018). Propriospinal neurons that control limb movements, posture, or autonomic functions are also found in the cervical and thoracic cord. Consequently, propriospinal neurons cannot be distinguished based on location alone. In fact, some propriospinal neurons may perform dual functions in respiration and limb movements (Le Gal et al., 2016). In addition, propriospinal neurons may influence respiratory centers *via* ascending projections to the brainstem (Lane et al., 2008; Jones et al., 2012). Thus, respiratory propriospinal neurons include a diverse group of neurons throughout the spinal cord that likely perform many distinct functions.

Propriospinal neurons likely help coordinate activity between different respiratory muscle groups, as upper cervical neurons have been described that control both phrenic and intercostal motor neurons (Lipski and Duffin, 1986). Further, dual tracing studies have described individual cervical neurons connected to both phrenic and intercostal motor neurons (Lane et al., 2008). Although bulbospinal neurons project throughout the spinal cord and can synchronize inspiratory motor neurons across multiple segments, propriospinal neurons appear to synchronize inspiratory motor neurons on a longer time scale and thus may provide an additional level of modulatory control (Kirkwood et al., 1982a,b). Likewise, although separate bulbospinal pathways provide the inspiratory and expiratory drive to respiratory motor neurons, propriospinal networks may provide reciprocal inhibition between inspiratory and expiratory intercostal motorneurons (Aminoff and Sears, 1971). These propriospinal networks may provide an additional level of control for modulation of breathing by sensory afferents or the cerebral cortex.

It has been hypothesized that propriospinal neurons can modify the amplitude of motor output by relaying and/or amplifying central respiratory drive to motor neurons. This is based on observations that at least a subset of propriospinal neurons are innervated by inspiratory rVRG neurons or display inspiratory-related phasic bursting (Hilaire et al., 1983, 1986; Palisses et al., 1989; Duffin and Iscoe, 1996; Hayashi et al., 2003; Lane et al., 2008; Sandhu et al., 2015). Multiunit recordings of spinal neurons and cross-correlation analyses have identified both excitatory and inhibitory pre-phrenic propriospinal neurons in the rat cervical cord (Sandhu et al., 2015; Streeter et al., 2017). Acute intermittent hypoxia (a known driver of respiratory plasticity) enhances functional connectivity between excitatory neurons and pre-phrenic propriospinal neurons, suggesting that changes in propriospinal neuron function can increase motor output (Streeter et al., 2017). In addition, inhibitory neurons help pattern the duration of motor output. Studies of the rat phrenic nucleus have identified inspiratory, expiratory, and tonic firing inhibitory (GABAergic) interneurons (Marchenko et al., 2015; Ghali, 2018). The inspiratory inhibitory neurons include, but are probably not limited to, Renshaw cells that provide recurrent inhibition to phrenic and intercostal motor neurons (Kirkwood et al., 1981; Hilaire et al., 1983, 1986; Lipski et al., 1985; Iizuka et al., 2018). Blocking GABAergic inhibition by spinal neurons alters the shape of the phrenic motor burst and increases activity during expiration (Marchenko et al., 2015). Thus, it is likely that spinal circuits shape the relatively uniform respiratory drive from the brainstem into a pattern of activity-specific for each motor pool, at least under some conditions.

Why do we need muscle-specific patterns of motor activity? One explanation is that propriospinal neurons help ensure that breathing is efficient by making effective use of body biomechanics, which can vary between species and even individuals. The timing, discharge frequency, and patterns of activity vary between inspiratory motor pools during breathing (Butler et al., 2014). It has been proposed that “neuromechanical matching” of the drive to the inspiratory motor neurons ensures the most efficient contraction of inspiratory muscles based on their mechanical advantage (De Troyer et al., 2005; Butler et al., 2014). In humans, for example, rostral intercostal muscles are preferentially recruited for inspiration because they provide a higher mechanical advantage compared to caudal intercostal muscles. This pattern cannot be attributed solely to intrinsic motor neuron properties and does not require afferent input. Moreover, this pattern is mediated by spinal circuits because the same pattern of intercostal muscle activity is seen following high-frequency electrical stimulation of the spinal cord in animals with a C2 spinal section as is observed in spontaneously breathing dogs (DiMarco and Kowalski, 2011). Neuromechanical matching of the inspiratory drive to respiratory muscles is likely mediated by excitatory propriospinal neurons because blocking inhibitory neurotransmission does not alter the rostrocaudal gradient of thoracic inspiratory motor activity in a neonatal rat spinal cord preparation (Oka et al., 2019). Because disease and injury can alter an individual’s breathing biomechanics, plasticity within propriospinal circuits is likely critical to maintaining efficient breathing.

### Propriospinal Neurons Process and Transmit Sensory Afferent Information

Respiratory muscle afferents influence multiple aspects of respiratory motor output as well as autonomic functions (for review, see Nair et al., 2017b). Phrenic afferents include group Ia (muscle spindle that sense stretch), Ib (Golgi tendon organs that sense tension/load), group III and IV afferents that sense metabolites/fatigue, and pressure-sensitive afferents in the diaphragm that might be Pacinian corpuscles (Nair et al., 2017b). Compared to limb or intercostal muscles, the diaphragm has a relatively low proportion of Ia afferents, but the significance of this difference for respiratory control is not clear (Corda et al., 1965; Road, 1990). It is important to note that proprioceptive reflexes mediated by muscle afferents (that project into the spinal cord) are distinct from the Hering-Breuer reflex mediated by vagal pulmonary stretch receptors that project to the brainstem. Proprioceptive information is used in many ways to control the pattern of breathing through local spinal circuits as well as spino-bulbar-spinal circuits.

Propriospinal neurons likely play a key role in the processing of afferent information and relaying it to the appropriate targets in the spinal cord and brainstem. In support of this hypothesis, phrenic afferents rarely project directly to phrenic motor neurons that control the diaphragm; instead, most project it to the interneurons in the dorsal or intermediate laminae (Nair et al., 2017a). Notably, the propriospinal neurons in the cervical cord that respond to phrenic afferent stimulation appear to be largely distinct from the neurons that receive rhythmic respiratory drive (Cleland and Getting, 1993; Iscoe and Duffin, 1996).

Respiratory afferents mediate both excitatory and inhibitory reflexes *via* spinal and supraspinal circuits (Gill and Kuno, 1963; Marlot et al., 1987; Macron et al., 1988). For example, stimulation of the phrenic nerve in various animal models can cause bilateral inhibition of the diaphragm (the phrenic-to-phrenic reflex; Gill and Kuno, 1963; Marlot et al., 1987; Speck and Revelette, 1987), as well as external intercostal muscles (the phrenic-to-intercostal reflex; Brichant and De Troyer, 1997; De Troyer, 1998; De Troyer et al., 1999). A spinal transection at C2 abolishes the contralateral phrenic-to-phrenic reflex, but the ipsilateral reflex is maintained (Speck, 1987). This result suggests that spinal circuits mediate ipsilateral inhibition of respiratory muscles but supraspinal circuits are involved in contralateral inhibition. However, it is not known whether the contralateral reflex is mediated by a spino-bulbar-spinal loop or whether supraspinal input is merely required to facilitate a spinal reflex, as has been demonstrated for group Ib and II afferent control of limb muscles (Cabaj et al., 2006). Phrenic afferents can also inhibit the diaphragm, intercostal and scalene muscles in humans and the latency suggests that it might involve brainstem circuits (Butler et al., 2003). The circuitry may involve multiple components as phrenic nerve stimulation can produce a biphasic response in which inhibition is followed by a long latency excitatory response (Marlot et al., 1987; Supinski et al., 1993). Moreover, the impact of afferents can be quite significant as high-intensity stimulation of phrenic group III–IV afferents can stimulate ventilation up to 4–5 times baseline values (Yu and Younes, 1999). Thus, the regulation of breathing by phrenic afferents is complex-involving multiple afferent types (and presumably spinal interneuron types) as well as both spinal and supraspinal circuitry.

Intercostal afferents sense changes in the muscles controlling the chest wall and can influence not only intercostal muscle activity, but also diaphragm and accessory respiratory muscle activity (Butler et al., 2014; McBain et al., 2016). Stimulation of intercostal afferents can either inhibit or facilitate phrenic motor activity and reflexes appear to be mediated by spinal (excitatory and inhibitory) and supraspinal (inhibitory) components (Decima et al., 1967; Decima and von Euler, 1969; Remmers, 1973; Bellingham, 1999). Short-latency intersegmental reflexes have also been described between intercostal and scalene muscles in humans (McBain et al., 2016). The significance of spinal intersegmental reflexes for coordinating respiratory muscle activity may best be illustrated by the observation that proprioceptive afferents can entrain phrenic nerve activity to chest movements driven by a ventilator even in ‘spinalized’ animals (Persegol et al., 1987).

Identifying the propriospinal and brainstem neurons that mediate the different types of afferent input and investigating how each of them influences respiratory motor activity could advance the development of diaphragm pacing or spinal stimulation devices to retore ventilation following disease or injury.

### Propriospinal Neurons Help Coordinate Ventilation and Motor Activity

During exercise, mammals increase ventilation to maintain proper arterial oxygen and carbon dioxide levels despite elevated metabolic activity (Forster et al., 2012; Guyenet and Bayliss, 2015). This is accomplished without directly sensing the rate of gas exchange in muscle or lungs. The mechanisms that lead to exercise hyperpnea are thought to include both a feed-forward central command mechanism (particularly at the onset of exercise) as well as group III/IV afferent feedback from limb muscles (Forster et al., 2012). Although the circuits that mediate these responses to match ventilation to motor activity are not currently known, it is likely that propriospinal neurons play critical roles. In addition to ensuring appropriate overall respiratory activity levels, propriospinal neurons are likely involved in the cycle-by-cycle coordination between locomotor and respiratory periods, which we refer to as entrainment.

There is mounting evidence that spinal respiratory circuits receive input from the locomotor central pattern generator (CPG). For example, activation of fictive locomotion in isolated brainstem/spinal cord preparations can cause an increase in phrenic burst frequency as well as entrainment of locomotor and respiratory periods (Le Gal et al., 2014; Yazawa, 2014). These responses are likely mediated in part by reciprocal connections between spinal and brainstem circuits. However, there are also intersegmental interactions within the spinal cord that link the locomotor and respiratory circuits. For example, phrenic motor neurons can be driven by lumbar spinal circuits when local inhibition is blocked even following a C1 transection (Cregg et al., 2017). In addition, there is entrainment (a 1:1 coupling between successive periods) between locomotor and phrenic motor output in spinalized rabbits (Viala et al., 1987). This entrainment might reflect direct interaction between the locomotor CPG and spinal circuits driving respiration (Viala, 1986). In fact, the division between “locomotor” and “respiratory” circuits might not be as precise as is usually assumed. Bimodal spinal neurons were recently described in a neonatal brainstem spinal cord preparation that were expiratory and also received flexor-related drive *via* propriospinal pathways (Le Gal et al., 2016). Additional research is needed to investigate the potential overlap between locomotor and respiratory neurons in the spinal cord and brainstem as well as their interconnections, particularly in adult animals.

Limb muscle afferents play important roles in matching locomotor and respiratory activity (exercise hyperpnea) as well as coupling the respiratory and locomotor cycles (entrainment; Shevtsova et al., 2019). However, as some afferents have direct projections to the brainstem, the role of propriospinal neurons in mediating these effects needs further investigation. For example, stimulation of limb muscles or nerves can increase phrenic activity *via* brainstem respiratory centers but can also inhibit phrenic activity *via* spinal circuits (Eldridge et al., 1981). *In vitro* preparations have been used to demonstrate that limb afferent stimulation affects the rate of ventilation (i.e., coordinating overall locomotor and respiratory activity levels) through its effects on spinal neurons of the locomotor CPG (Morin and Viala, 2002). On the other hand, entrainment of the locomotor and respiratory periods appears to occur *via* ascending limb afferent projections directly to brainstem respiratory centers (Morin and Viala, 2002; Giraudin et al., 2008, 2012). This is consistent with experiments showing that stimulation of limb muscle afferents and phrenic afferents can increase ventilation in an additive manner, suggesting they act through different mechanisms (Ward et al., 1992). The fact that spinalized rabbits show locomotor-respiratory entrainment after treatment with nialamide and DOPA suggests that multiple mechanisms of entrainment may exist (Viala, 1986). These experiments suggest that limb afferent stimulation and/or limb motor rehabilitation could improve breathing following disease or injury.

### Propriospinal Neurons Regulate Accessory Respiratory Muscle Activity

Accessory respiratory muscles enhance ventilation under conditions of increased oxygen demand. Inspiratory accessory respiratory muscles are used to increase thoracic volume to ensure sufficient ventilation and include the scalene, trapezius, pectoralis, sternocleidomastoid and parasternal muscles (Sieck and Gransee, 2012). Like the diaphragm and external intercostal muscles, accessory respiratory muscles receive rhythmic drive from the ventral respiratory group of the medulla, but additional pathway(s) may also contribute to activating accessory respiratory muscles at the onset of exercise or following disease or injury (De Troyer et al., 2005; Butler, 2007; Johnson and Mitchell, 2013; Butler et al., 2014). Expiratory accessory respiratory muscles include the internal intercostals and abdominal muscles. In healthy humans, accessory respiratory muscles help stabilize the thoracic cavity during eupnea and enhance ventilation during conditions of high oxygen demand, such as exercise (Sieck and Gransee, 2012; Aliverti, 2016). Accessory respiratory muscles are also used to increase ventilation following neuromuscular disease and injury (Johnson and Mitchell, 2013).

Recent studies have implicated propriospinal circuits in the recruitment of accessory respiratory muscles for breathing. Romer et al. (2017) showed that chemogenetic activation of V2a neurons is sufficient to activate scalene and trapezius accessory respiratory muscles and increase ventilation. A subsequent study showed that chemogenetic inhibition of V2a neurons also surprisingly activated scalene and trapezius accessory respiratory muscles (Jensen et al., 2019). Since accessory respiratory muscle activation was not accompanied by impaired diaphragm activity, it was postulated that a subset of V2a neurons (presumably different from the subset that activates inspiratory accessory respiratory muscles) participates in an inhibitory pathway to prevent accessory respiratory muscle activation at rest, when they are not needed. However, these studies did not distinguish whether spinal or brainstem V2a neurons are responsible for activating or inhibiting accessory respiratory muscles. Additional studies are necessary to further elucidate the role of different propriospinal neurons in controlling accessory respiratory muscle activity.

Propriospinal neurons serve important roles in patterning respiratory muscle activity to promote efficient and effective ventilation, even during exercise or following disease and injury. A better understanding of these pathways could aid in the development of therapies to improve breathing when pathways to/from the brainstem are disrupted following SCI.

## Roles of Propriospinal Neurons in Recovery of Respiratory Motor Function After Injury

In the following sections, we describe evidence that propriospinal circuits involved in breathing undergo significant anatomical and functional changes following injury. We provide evidence that these changes significantly contribute to the recovery of respiratory function that can be seen following SCI. Moreover, we suggest that therapies targeting propriospinal neurons may improve the recovery of breathing in patients failing to recover on their own.

### Propriospinal Neurons Show Altered Anatomical and Functional Connectivity to Respiratory Motor Neurons Following Injury

Propriospinal neurons show anatomical and functional changes that suggest they contribute to the spontaneous recovery of respiratory function following SCI. For example, despite the presence of inhibitory molecules, cervical spinal commissural interneurons show substantial axonal regeneration across the midline of the cat spinal cord following a midsagittal axotomy without any therapeutic intervention (Fenrich and Rose, 2009). Moreover, these commissural interneurons form functional synapses with motor neurons within 2–3 months, as confirmed by electrophysiology (Fenrich and Rose, 2009). Uninjured propriospinal neurons below the site of an injury may also undergo changes in connectivity. For example, the connectivity between spinal V2a neurons and phrenic motor neurons is increased in rats 2 weeks following a C2 hemisection injury (Zholudeva et al., 2017). Spinal plasticity is not unique to respiratory interneurons, as the emergence of novel intraspinal circuits to enhance motor behavior has been reported across multiple levels of the spinal cord (Bareyre et al., 2004; Courtine et al., 2008; Filli and Schwab, 2015).

Functional changes within propriospinal circuits both above and below a SCI are likely important for recovery from injury. Caudal to a C2 hemisection injury, excitatory connections between propriospinal neurons across the cord show a bias for the contralateral-to-ipsilateral direction, suggesting that changes in spinal circuits may help relay drive to neurons deprived of input following injury (Streeter et al., 2020). Changes in propriospinal circuits also occur above a spinal cord lesion. For example, expiratory bulbospinal neurons make more functional projections to propriospinal neurons (but not motor neurons) in the thoracic cord above an injury (Ford et al., 2016). Taken together, these studies suggest that propriospinal neurons may be important mediators of respiratory plasticity following SCI. However, additional studies are necessary to investigate how the plasticity of respiratory circuits can be leveraged to further improve breathing following injury.

### Propriospinal Neurons Modulate Respiratory Motor Output Following Spinal Cord Injury

Spinal stimulation studies have demonstrated the importance, as well as the potential, of spinal circuits to drive breathing following SCI. Studies from dogs and rats completely transected at the cervical level show that high-frequency spinal cord stimulation on the ventral surface at thoracic levels activates both intercostal and diaphragmatic muscles, as well as enhances inspired volume (DiMarco and Kowalski, 2009, 2011, 2019; Kowalski et al., 2013). This stimulation acts through propriospinal neuron networks rather than descending brainstem input, as it is effective 5 days after a complete trans-section when bulbospinal axons have already degenerated (DiMarco and Kowalski, 2019). Moreover, the specific pattern of respiratory muscle activity is the same after transection as it was prior to the injury, demonstrating the importance of spinal circuits for patterning activity (DiMarco and Kowalski, 2011). High-frequency spinal cord stimulation has also successfully been used to restore cough in patients with SCI (DiMarco et al., 2014). Direct intraspinal stimulation has also been used in rats to activate phrenic motor neurons for breathing, likely *via* propriospinal neurons (Mercier et al., 2017). However, the neural substrates upon which the spinal cord stimulation acts are not currently clear.

Additional evidence that spinal circuits are sufficient to drive and sustain breathing comes from studies using optogenetics to alter activity in cervical neurons (Alilain et al., 2008). Alilain et al. (2008) were able to restore activity to the ipsilateral diaphragm after a C2 hemisection by exciting channel rhodopsin expressing neurons at the level of the phrenic nucleus (which included phrenic motor neurons as well as excitatory and inhibitory propriospinal neurons) with light. In addition to direct activation of phrenic motor activity with light stimulation, they also observed a rhythmic waxing and waning of rhythmic bursting activity even after cessation of photostimulation, which was hypothesized to result from spinal network activity. These results suggest that spinal respiratory networks may exhibit central pattern generator properties, at least under some conditions.

Maintenance of respiratory function following injury is likely dependent on excitatory propriospinal neurons. In a mouse model of non-traumatic SCI, respiratory function is maintained despite substantial damage to spinal tracts and phrenic motor neuron loss, similar to most human patients with cervical myelopathy. However, silencing glutamatergic propriospinal neurons results in impaired breathing in injured, but not healthy mice (Satkunendrarajah et al., 2018). These results indicate that propriospinal neurons normally help sustain breathing following injury. Further, increasing the activity of glutamatergic neurons can also restore function to the diaphragm following an acute C2 hemisection injury (Satkunendrarajah et al., 2018). Thus, increasing the activity of excitatory propriospinal neurons holds promise as a therapy to improve breathing following traumatic or non-traumatic injuries.

Inhibitory propriospinal neurons are also important modulators of respiratory function following SCI. For example, blocking the effects of inhibitory interneurons using a GABA receptor antagonist can restore rhythmic bursting activity to a paralyzed hemidiaphragm below a C2 hemisection injury (Zimmer and Goshgarian, 2007). In addition, Cregg et al. (2017) used an *ex vivo* neonatal spinal cord preparation to show that blocking inhibitory neurons could elicit phrenic motor neuron bursting even after complete C1 transection. Optogenetic stimulation of excitatory neurons could evoke phrenic bursts in their preparation, but only when inhibitory neurotransmission was blocked (Cregg et al., 2017). Thus, phrenic motor neurons appear to be targets of excitatory pathways that are normally latent due to the activity of inhibitory neurons. These results are consistent with studies of locomotor circuits that demonstrate that inhibitory neurons can act as critical obstacles of functional recovery after SCI and that targeting these neurons can improve recovery (Chen et al., 2018).

Several groups have investigated the potential of induced pluripotent stem cell (iPSC) derived propriospinal neurons as a potential therapy to restore respiratory motor function. For example, iPSC derived V2a and V3 neurons have been generated from mouse or human cells (Brown et al., 2014; Xu et al., 2015; Iyer et al., 2016; Butts et al., 2017, 2019; White and Sakiyama-Elbert, 2019). iPSC derived V2a neurons have been transplanted into the spinal cord where they survive and appear to form synapses with host neurons (Butts et al., 2017; Zholudeva et al., 2018a). Moreover, iPSC derived V2a neurons appear to benefit recovery because rats who received a spinal injection of V2a neurons along with neural progenitor cells showed a greater recovery of diaphragm function 1 month after a C2 hemisection than rats who received neural progenitor cells alone (Zholudeva et al., 2018a). Thus, both intrinsic and extrinsic propriospinal neurons may be able to promote recovery of breathing following injury.

## Using Developmental Markers to Target Propriospinal Neuron Classes and Test Their Roles in Breathing

A better understanding of which propriospinal neurons are important for breathing and how they contribute to the recovery of function will be vital for developing treatments. For example, therapies that target all excitatory and/or all inhibitory neurons in the spinal cord could produce unwanted side effects in SCI patients, including spasticity, chronic pain, and autonomic dysregulation. Here, we provide some evidence that developmental spinal neuron classes play distinct roles in locomotor and respiratory circuits as well as speculate on potential functions of these neurons in cases where a role in breathing is not yet known.

### Developmental Neuron Classes Perform Distinct Functions

Molecular genetic approaches to label specific developmental classes of spinal neurons have allowed investigators to begin dissecting the complex circuitry of the spinal cord (Grillner and El Manira, 2015; Lu et al., 2015; Kiehn, 2016; Gosgnach et al., 2017; Ziskind-Conhaim and Hochman, 2017; Dougherty and Ha, 2019). Developmental studies have identified 10 progenitor domains that give rise to six dorsal (dI1–6) and four ventral (V0–V3) classes of propriospinal neurons. Characterization of the progenitors and/or the post-mitotic neurons derived from each domain has identified specific molecular markers (i.e., transcription factors) that distinguish each class of neurons. Each class is found throughout the spinal cord from cervical to sacral segments. A similar pattern of transcription factor expression is also found during the development of the brainstem, giving rise to comparable broadly defined neuron classes (Gray, 2013). Roles for each neuron class in the control of locomotion have been investigated through experiments in which the development, survival, or function of a specific class of neurons is altered while assessing locomotor function. From these studies, it has become apparent that the different developmental subclasses of propriospinal neurons have different properties (i.e., neurotransmitter identity, projection pattern, presynaptic inputs) and perform different roles in locomotion (Lu et al., 2015; Ziskind-Conhaim and Hochman, 2017).

**Figure 2:**
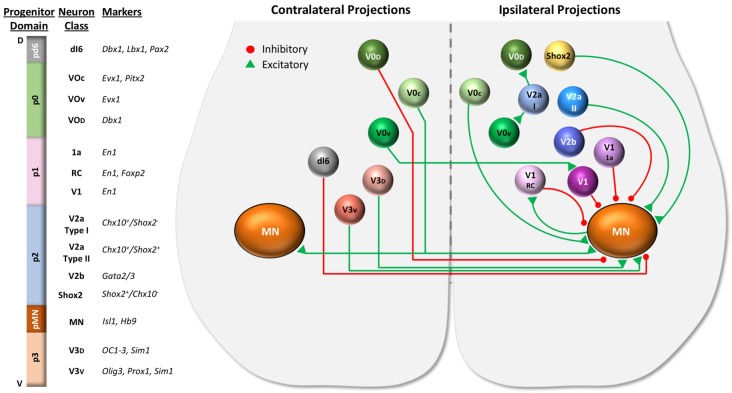
Spinal cord progenitor domains give rise to distinct developmental neuron classes. Distinct progenitor domains organized along the dorsal (D) to ventral (V) axis give rise to distinct classes of neurons. Shown on the left are progenitor domains located in the ventral portion of the spinal cord, including the motor neuron progenitor (pMN) domain, four ventral progenitor domains (p0–p3), and one dorsal progenitor domain (pd6), along with the neuron classes they give rise to and molecular markers used to identify the neuron classes derived from each domain. The dorsal progenitor domains pd1–5 are not shown. The diagram of the ventral spinal cord on the right illustrates some of the properties (excitatory vs. inhibitory, ipsilateral vs. contralateral projections, etc.) as well as the interactions between ventral neuron classes. Commissural propriospinal neurons (shown on the left side of cord) project contralaterally across the midline to contact neurons on the opposite side of the spinal cord. These propriospinal neurons include dI6, V0, and V3 classes. Ipsilaterally projecting propriospinal neuron classes are shown on the right side of the cord. The broad neuronal classes may be further divided into subclasses. For example, the V1 class includes Renshaw cells (V1 RC), Ia inhibitory neurons (V1 1a), and other inhibitory neurons (V1). V2 neurons can be divided into Chx10+ V2a neurons, Gata 2/3+ V2b neurons, and Shox2 (non-Chx10+) neurons. Chx10 expressing V2a neurons can be further divided based on Shox2 expression into type I (V2a I) and type II (V2a II) neurons.

A summary of the properties and connectivity of the major developmental neuron classes in the ventral part of the spinal cord is shown in Figure 2. Most of the data in this figure is based on studies of locomotor circuits in the lumbar spinal cord (Lu et al., 2015; Ziskind-Conhaim and Hochman, 2017). However, it is likely that many of the properties and functions of these neurons are conserved in respiratory circuits, but with some important differences. For example, V2a and V0 neurons work together to ensure that the left and right limbs alternate during locomotion. Efficient breathing, however, requires synchronous inspiration and expiration on the left and right sides. V0 neurons (most likely in the brainstem) are critical for this coordination because disruption of commissural projections of V0 neurons results in left-right desynchronized inspiration (and neonatal lethality; Wu et al., 2017). Thus, similar neural building blocks may be used in different ways in locomotor vs. respiratory circuits.

Importantly, each broad developmental class of neurons can be further subdivided into multiple subtypes that likely play distinct roles in motor behaviors. For example, the V1 class includes Renshaw cells, Ia inhibitory neurons, as well as additional inhibitory neuron classes (Alvarez et al., 2005). In fact, detailed gene expression analyses can divide V1 neurons into over 50 inferred neuron subtypes and V2a neurons into at least 11 subtypes (Bikoff et al., 2016; Gabitto et al., 2016; Hayashi et al., 2018; Sweeney et al., 2018). Once markers of specific subtypes are identified, intersectional genetic tools may be used to elucidate specific roles for different neural subtypes in breathing or other motor functions (Ray et al., 2011; Brust et al., 2014; Hennessy et al., 2017).

### Potential Roles for Developmental Neuron Classes in Breathing

Many, if not all, of the cardinal developmental neuron classes, are likely to play a role in the control of breathing. Genetic tools to target subsets of these neurons may help to elucidate the roles of these neurons in respiratory circuits as well as help identify the cellular targets most likely to improve breathing following injury. For example, Wu et al. (2017) used a combination of viral tracing and transgenic labeling methods to show that brainstem neurons of the V0 class in the PBC and rVRG are critical components of the circuits driving inspiration. Moreover, they were able to disrupt the development of these neurons to demonstrate the importance of commissural projections for the synchronous activity of the left and right side of the diaphragm.

In locomotor circuits, V1 neurons shape motor bursts to control the duration of stance and swing phases of the step cycle and thus the speed of walking (Gosgnach et al., 2006). Renshaw cells (a subset of V1 neurons) provide recurrent inhibition to phrenic and intercostal motor neurons (Kirkwood et al., 1981; Hilaire et al., 1983, 1986; Lipski et al., 1985; Iizuka et al., 2018), but the role of most V1 neurons in breathing is unknown. One could hypothesize that the GABAergic neurons that shape respiratory motor bursts (Marchenko et al., 2015) are a subset(s) of V1 neurons. Experiments in which V1 neurons are optogenetically activated or silenced could test the significance of inhibitory modulation from V1 neurons on respiratory motor output *in vitro*, or even in healthy or injured animals. In addition, the role of proprioceptive afferents in the control of breathing could be further probed by mapping their targets in the spinal cord and brainstem (Nair et al., 2017a) and manipulating the activity of each of their targets. These targets are likely to include subsets of V1 neurons (e.g., Ia and Ib inhibitory neurons), but may also include additional neuron types.

In locomotor circuits, V2a neurons are important for speed-dependent control of gait (left-right alternation of limbs) as well as the regularity of motor bursts (Crone et al., 2008, 2009). V2a neurons in the brainstem appear to be important for generating regular, frequent breathing in neonatal mice (Crone et al., 2012), but are dispensable for breathing in adult mice at rest (Jensen et al., 2019). In adult mice, V2a neurons appear to be particularly important for the control of accessory respiratory muscles. For example, there appear to be at least two subsets of V2a neurons in the cervical cord, one subset that activates accessory inspiratory muscles at rest (Romer et al., 2017) and another subset that prevents their activation at rest when they are not needed (Jensen et al., 2019; see “Propriospinal Neurons Regulate Accessory Respiratory Muscle Activity” section). These results suggest that a major role for V2a neurons may be in matching recruitment of accessory respiratory muscles to levels of motor activity, but this hypothesis has not yet been directly tested.

The major role of V3 neurons in locomotor circuits is to balance excitation across the left and right sides of the cord (Zhang et al., 2008). Although commissural projections of V0 bulbospinal neurons synchronize the inspiratory drive between the left and right sides (Wu et al., 2017), one could speculate that V3 neurons also play a role in balancing the activity of respiratory circuits across the cord that may be particularly important in the context of injury when damage may be more severe on one side of the cord (e.g., the circuit changes described by Streeter et al., 2020). An additional potential role for V3 neurons in breathing includes transmitting afferent input to respiratory motor neurons or propriospinal neurons. For example, V3 neurons mediate sensory-evoked muscle spasms of tail muscles following SCI (Lin et al., 2019). Additional research is necessary to identify the roles of V3 neurons in respiratory circuits as well as their potential roles in plasticity following injury.

## Conclusions

There is convincing evidence that propriospinal neurons help pattern the activity of respiratory muscles in order to meet the needs of the organism. This is a dynamic process that involves a variety of neurons with unique roles. In SCI, the need to modulate motor function is escalated and the roles of propriospinal neurons in the control of breathing may be amplified. Indeed, there is mounting evidence that spinal circuitry is altered after disease and injury, that spinal network plasticity contributes to recovery, and that propriospinal neurons are an attractive therapeutic target to improve respiratory motor function. Developmental markers that can be used to identify and genetically mark (or manipulate) specific classes of propriospinal neurons have been valuable tools to investigate the function of neurons within circuits. These tools will continue to be useful as we investigate mechanisms of neuroplasticity that promote recovery of function following injury. Overall, a better understanding of the circuitry that controls breathing in uninjured animals and the changes that occur following injury should lead to new therapies to improve breathing.

## Author Contributions

SC, WA, and VJ wrote the article.

## Conflict of Interest

The authors declare that the research was conducted in the absence of any commercial or financial relationships that could be construed as a potential conflict of interest.
